# Human rhinoviruses and enteroviruses in influenza-like illness in Latin America

**DOI:** 10.1186/1743-422X-10-305

**Published:** 2013-10-11

**Authors:** Josefina Garcia, Victoria Espejo, Martha Nelson, Merly Sovero, Manuel V Villaran, Jorge Gomez, Melvin Barrantes, Felix Sanchez, Guillermo Comach, Ana E Arango, Nicolas Aguayo, Ivette L de Rivera, Wilson Chicaiza, Mirna Jimenez, Washington Aleman, Francisco Rodriguez, Marina S Gonzales, Tadeusz J Kochel, Eric S Halsey

**Affiliations:** 1 US Naval Medical Research Unit 6, Lima, Peru; 2 Fogarty International Center, National Institutes of Health, Bethesda, MD, USA; 3 Dirección General de Epidemiología, Ministerio de Salud, Lima, Perú; 4 Hospital Solano, Buenos Aires, Argentina; 5 Hospital Infantil Manuel de Jesus Rivera, Managua, Nicaragua; 6 LARDIDEV-Biomed-UC, Maracay, Venezuela; 7 Universidad de Antioquia, Medellín, Colombia; 8 ONG Rayos de Sol, Asuncion, Paraguay; 9 Universidad Nacional Autónoma de Honduras, Tegucigalpa, Honduras; 10 Hospital Vozandes and Universidad de las Americas, Quito, Ecuador; 11 Hospital Nacional de Metapan, Metapan, El Salvador; 12 Clinica Alcivar and Hospital Vernaza, Guayaquil, Ecuador; 13 Hospital Departamental Humberto Alvarado de Masaya, Masaya, Managua, Nicaragua; 14 Laboratorio Departamental, Secretaria Seccional de Salud del Meta, Villavicencio, Colombia; 15 US Naval Medical Research Center, Silver Spring, MD, USA

## Abstract

**Background:**

Human rhinoviruses (HRVs) belong to the *Picornaviridae* family with high similarity to human enteroviruses (HEVs). Limited data is available from Latin America regarding the clinical presentation and strains of these viruses in respiratory disease.

**Methods:**

We collected nasopharyngeal swabs at clinics located in eight Latin American countries from 3,375 subjects aged 25 years or younger who presented with influenza-like illness.

**Results:**

Our subjects had a median age of 3 years and a 1.2:1.0 male:female ratio. HRV was identified in 16% and HEV was identified in 3%. HRVs accounted for a higher frequency of isolates in those of younger age, in particular children < 1 years old. HRV-C accounted for 38% of all HRVs detected. Phylogenetic analysis revealed a high proportion of recombinant strains between HRV-A/HRV-C and between HEV-A/HEV-B. In addition, both EV-D68 and EV-A71 were identified.

**Conclusions:**

In Latin America as in other regions, HRVs and HEVs account for a substantial proportion of respiratory viruses identified in young people with ILI, a finding that provides additional support for the development of pharmaceuticals and vaccines targeting these pathogens.

## Background

Acute respiratory infections (ARIs) are a leading cause of acute illness worldwide and remain the most important cause of pediatric mortality [1]. Lower respiratory tract infections (LRTIs) are among the leading causes of hospitalization and death in children less than 5 years old worldwide, particularly in resource-poor countries [2].

Human rhinoviruses (HRVs) and enteroviruses (HEVs) belong to the *Picornaviridae* family and are prominent causes of respiratory disease [3]. They share identical genomic organization and high sequence homology [4]. Their genome is divided into three sections: a 5’untranslated region (5’UTR), an open reading frame of the polyprotein that codes for all four capsid proteins (VP1-4) and the non-structural genes, and a 3’untranslated region [5].

Although many reports link HRV primarily to illness in children [6,7], disease in other populations such as military recruits [8] and nursing home residents have been reported [9]. HRV infection often results in mild upper respiratory disease like the common cold, but it may also cause more serious disease by exacerbating asthma or other pre-existing respiratory disorders. In contrast, HEVs infect primarily the gastrointestinal tract and can spread to other sites, but some HEVs display specific tropism for the respiratory tract [10,11].

There are more than 100 different serotypes of HRVs taxonomically grouped into two species HRV-A and HRV-B, according to the alignment of nucleotide fragments of the VP1 gene, the VP4/VP2 gene, and, more recently, the whole genome sequence [4,12]. A different species, HRV-C, that shares 53 - 57% homology at the amino acid level with HRV-A and HRV-B, was identified in 2006 in patients with acute LRTIs in Africa, Asia, Australia, Europe and North America [13-16]. Since its detection, HRV-C has been reported to be a prominent respiratory pathogen in children, causing up to 5% of LRTIs [17] and found in 42% of children with influenza-like infection (ILI) without identification of another pathogen by conventional means [18]. HRV-C has also been implicated as a frequent cause of asthma exacerbation in children [15]. Recent studies indicate that HRVs, as well as HEVs, show great genetic diversity by recombination [17,19], as has been increasingly reported for the HRV-A/HRV-C recombinants [20].

There are few reports about HRVs and HEVs as causes of respiratory disease in Latin America [21-25], including one that describes HRV antibodies in Amazon tribes [26], but no large-scale genetic characterization of HRVs and HEVs has been performed in the region to date. In this study, we investigated the recent circulation of HRV and HEV with emphasis on recombinant strains in children and young adults in Latin America.

## Results

### HRV and HEV in Central and South America

**Figure 1:**
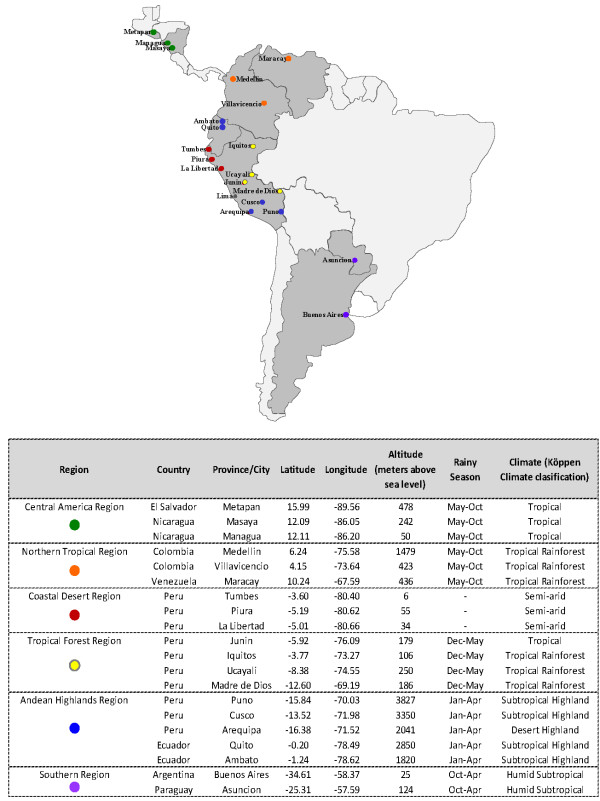
**Sample collection sites grouped by climatic/geographic similarities.** Most of the sample-collection sites (dots) throughout Latin America were grouped into six regions (colors) by their climatic and geographic similarities: latitude, longitude, altitude (meters above sea level), rainy season, and the Köppen climate classification were considered. Lima, Peru, was not considered for the temporal distribution analysis because it could not be grouped in to one of the six regions.

We collected 3,375 nasopharyngeal swabs from subjects with ILI symptoms from eight countries throughout Central and South America. We performed direct RT-PCR for HRVs and HEVs and sequenced all positive samples (n = 632) (Figure 1). Our subjects had a median age of 3 years, ranging from less than 1 month to 25 years, an interquartile range of 1 to 8 years, and a male/female ratio of 1.2:1.

**Figure 2:**
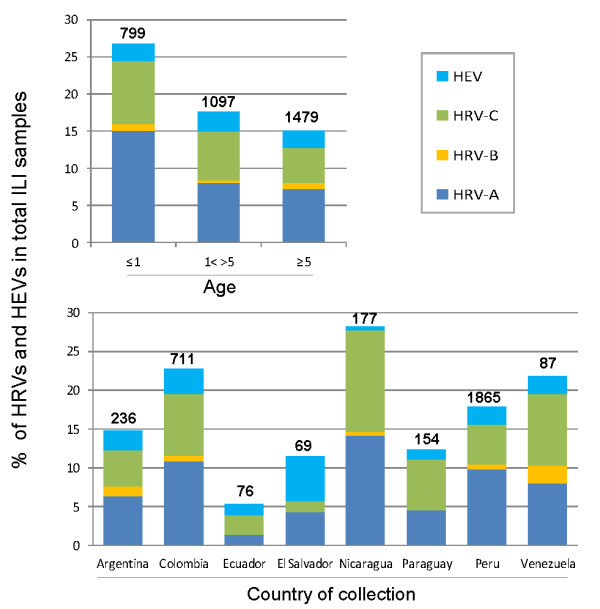
**Percentage of HRV and HEV by age and by country.** The percentage of human enteroviruses (HEV) and of each human rhinovirus species (HRV-A, HRV-B, and HRV-C) in samples from subjects with influenza like illness is shown by age (upper panel) and by country (lower panel). The total number of samples collected for each age group and country is shown above each percentage bar (**bold**).

Overall, HRVs and HEVs were identified in 16% (548 samples) and 3% (84 samples) of the ILI cases, respectively. Among the HRVs, HRV-A was the most represented species (9% of ILI cases), followed by HRV-C (6%) and HRV-B (1%). Although the number of ILI samples collected among countries varied considerably (Figure 2, lower panel) we found no statiscally significant geographic differences in the proportions of HEV, HRV-A, HRV-B, and HRV-C.

HRVs were identified significantly more frequently in children younger than 1 year (24%) compared to those between 1 and 5 years of age (15%) or older than five years (13%) (Figure 2, upper panel), a statistically significant finding (*p* <0.05 for both). However, using the two proportion z-test, we noted no difference in proportions of specific HRV species or HEVs per total ILI cases among different age groups. Nevertheless, the risk of detecting HRV-C in children younger than 5 years with ILI was 1.59 (C.I. 1.17-2.17; *p* <0.05) compared to older children and young adults (5–25 years).

Certain pre-existing respiratory conditions, such as rhinitis or chronic bronchitis, were found more often in those with HRV isolated (O.R. = 1.14 [95% C.I. 1.09 – 1.81]; *×*^2^ = 7.82; p-value < 0.05). Furthermore, the presence of these pre-existing conditions specifically increased the risk for detection of HRV-C (O.R. = 1.71 [95% C.I. 1.18 – 2.45]; *×*^2^ = 9.17; p < 0.05); asthma was a condition that doubled the risk of HRV-C detection (O.R. = 2.07 [95% C.I. 1.08 – 3.79]; *×*^2^ = 6.19; p <0.05).

**Figure 3:**
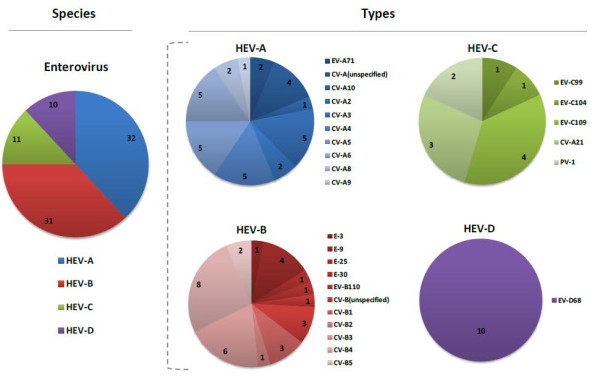
**Number of human enteroviruses detected.** Number of ILI samples (n = 84) in which human enteroviruses (HEVs) were detected divided into species (A-D) and for each species divided into types (EV = enterovirus, CV = coxsackievirus, E = echovirus, and PV = poliovirus).

Coxsackieviruses comprised the majority of the HEV group (65% of the HEVs identified), showing a variety of types: 9 for coxsackievirus A and 5 for coxsackievirus B (Figure 3). In addition, we detected multiple HEV serotypes, including EV-D68, EV-C99, EV-C104, EV-C109, and EV-B110. We also identified EV-A71 in two participants, both from the department of Tumbes, Peru, although from different cities. The first subject, a one year-old girl from the city of Tumbes (same name as the department), presented on April 27, 2011, with fever, rhinorrhea, cough, and erythema on pharyngeal examination. The second, a two year-old girl from the city of Zarumilla, presented on May 10, 2011, with fever, malaise, rhinorrhea, cough, and weight loss. Neither subject had rash, gastrointestinal manifestations, convulsions, change in consciousness, or other neurological deficits. Lastly, two polioviruses were detected and were related to the Sabin-1 polio vaccine strain.

**Figure 4:**
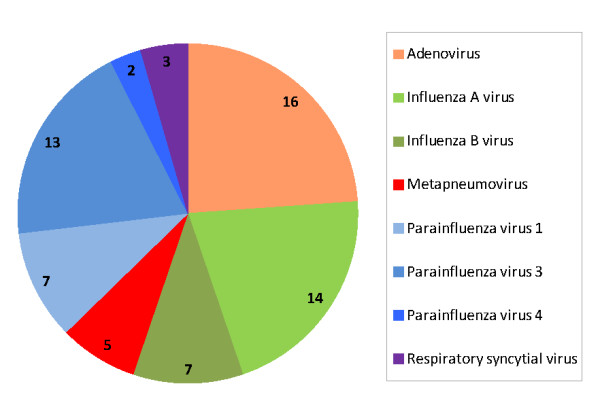
**Second virus detected in HRV/HEV positive samples.** Number of ILI samples (n = 67) where a second virus was detected in HRV-positive and HEV-positive samples.

By cell culture/immunofluorescence (as well as real time PCR for influenza viruses), we detected other respiratory viruses in 11% of the HRV-positive samples and 11% of the HEV-positive samples. The most commonly detected viruses were adenovirus, influenza virus A and parainfluenza virus 1 (Figure 4).

### Clinical manifestations of HRV and HEV infections

Although we found a higher frequency of cough, rhinorrhea, and dyspnea in subjects with HRV-C compared to those with etiologies other than HRV and HEV, this finding was not significantly more common when comparing HRV-C with HRV-A or HRV-B. HEV showed a significantly lower frequency of cough than HRV-C and the non-HRV/non-HEV group. Finally, there was no statistically significant difference in the rate of hospitalization for HRV-C (20%) compared to all other viruses. Other frequencies and comparisons are on Table 1.

**Table 1:** Clinical manifestations of HRV and HEV

	**Patient group**					***P*value for:**				
	**Other**	**HRV**			**HEV**	**C vs. other**	**C vs. A**	**C vs. B**	**C vs. HEV**	**HEV vs. other**
	**(n = 2810)**	**(n = 490)**			**(n = 75)**					
		**A**	**B**	**C**						
		**(n = 278)**	**(n = 20)**	**(n=192)**						
*General symptoms*										
Malaise	2080 (74.0)	193 (69.4)	18 (90.0)	130 (67.7)	55 (73.3)	0.11	0.74	0.05	0.45	0.91
Headache	1177 (41.9)	80 (28.8)	10 (50.0)	56 (29.2)	37 (49.3)	**0.06**	0.96	0.19	**>0.05**	0.37
Muscular pain	760 (27.0)	57 (20.5)	6 (30.0)	27 (14.1)	23(30.7)	0.14	0.48	0.35	0.16	0.69
Chills	353 (12.6)	29 (10.4)	4 (20.0)	22 (11.5)	13 (17.3)	0.88	0.9	0.64	0.63	0.62
*Respiratory symptoms*										
Cough	2558 (91.0)	260 (93.5)	20 (100.0)	183 (95.3)	61 (81.3)	**> 0.05**	0.42	0.32	**0.0006**	**0.009**
Rhinorrhea	2260 (80.4)	238 (85.5)	18 (90.0)	168 (87.5)	56 (74.7)	**0.02**	0.56	0.76	**0.02**	0.33
Sore throat	1588 (56.5)	147 (52.9)	13 (65.0)	88 (45.8)	44 (58.7)	**> 0.05**	0.29	0.19	0.16	0.77
Expectoration	814 (29.0)	83 (29.9)	7 (35.0)	64 (33.3)	17 (22.7)	0.47	0.66	0.93	0.4	0.57
Dyspnea	414 (14.7)	56 (20.1)	3 (15.0)	51 (26.6)	10 (13.3)	**0.03**	0.43	0.66	0.37	0.9
*Gastrointestinal symptoms*										
Vomiting	718 (25.6)	61 (21.9)	4 (20.0)	48 (25.0)	18 (24.0)	0.93	0.7	0.82	0.93	0.88
Abdominal pain	472 (16.8)	35 (12.6)	4 (20.0)	27 (14.1)	18 (24.0)	0.71	0.86	0.76	0.39	0.43
Diarrhea	349 (12.4)	50 (18.0)	5 (25.0)	20 (10.4)	12 (16.0)	0.79	0.43	0.39	0.64	0.71
Nausea	289 (10.3)	30 (10.8)	0 (0.0)	22 (11.5)	8 (10.7)	0.86	0.94	----	0.95	0.97
*Hospitalization*	295 (10.5)	36 (12.9)	2 (10.0)	38 (19.8)	6 (8.0)	0.09	0.42	0.73	0.49	0.84

The number of subjects presenting with each symptom is shown and the corresponding percentage is in parentheses on the right. “Other” represents ILI subjects that did not have HEVs or HRVs detected. HRV-C has been singled out to better show the comparison to other HRV species and other viruses. P-values for single analysis of each group--Other, HRV-A, HRV-B, HRV-C and HEV--are provided in the last columns. Only p-values <0.05 (**bold**) were considered statistically significant.

### Temporal distribution of HRV and HEV

**Figure 5:**
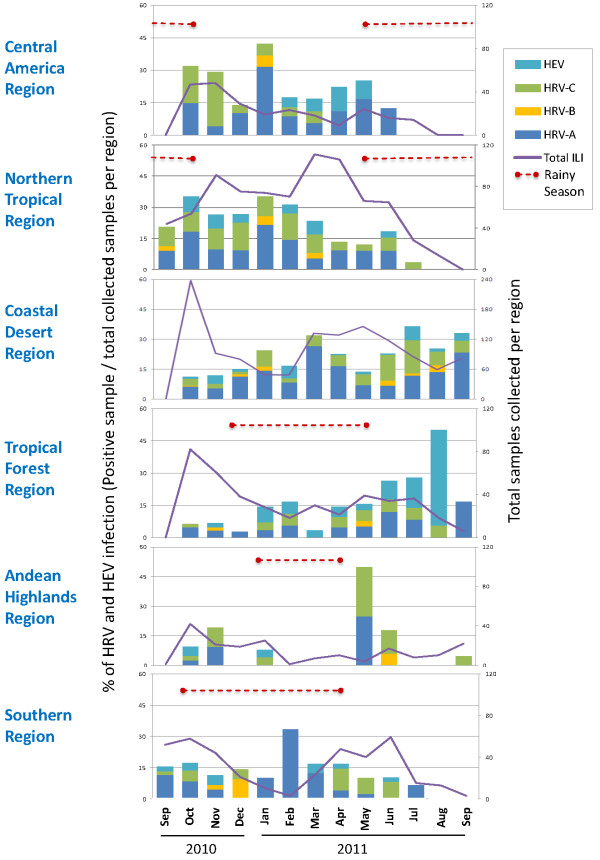
**Temporal distribution of HRV and HEV infections by region.** The percentage of viral infections (positive samples/total collected ILI samples per region in Figure 4) detected monthly is shown for HEV and each HRV species. The total ILI samples collected per month in each region is represented by the continuous purple line. The rainy season (RS) is depicted by a red dotted line.

We analyzed the temporal distribution of the three HRV species and HEV across six regions, grouped by similar climatic and geographical characteristics. Figure 1 shows the collection sites that were selected and grouped into regions for this analysis. Figure 5 depicts the percentage of each HRV species and HEV per total ILI samples collected and shows that HRV was present in tropical regions (Central America, northern and tropical forest regions) all year long. In no region was HRV or HEV activity detected more often in the rainy season or the higher temperature season. However, HRV-C accounted for a higher percentage (in most cases > 5%) of ILI cases north of the equator (first two panels) over the months of September 2010 to January 2011 and dropped in the following months, while the opposite was seen for the sites south of the equator (last four panels) where the detection of HRV-C increased during the months of April 2011 to July 2011.

### Phylogenetic analyses of HRV and HEV in Latin America

**Figure 6:**
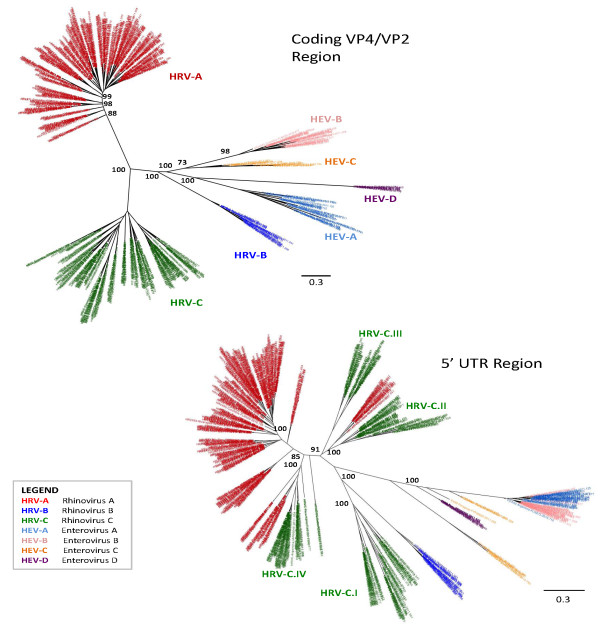
**Phylogenetic analyses of HRV and HEV.** This illustrates that recombination events are much more prevalent in the untranslated regions (UTRs) compared with a translated region (VP4/VP2). Separate alignments of the coding (VP4/VP2; 464 nt) and untranslated (5’UTR; 555 nt) regions’ sequences were constructed using MUSCLE v.3.8.31. Maximum likelihood phylogenetic trees were inferred separately for the non-coding and coding regions using PhyML v.3.0 using a general-reversible substitution model with gamma-distributed among-site rate variability. Samples are labeled by following format: “Sample code / Country of collection / Month- Year of collection.” Phylogenetic trees were colored by HRV species: HRV-A, HRV-B, HRV-C, and four types of HEV (HEV-A, HEV-B, HEV-C and HEV-D). In addition, four HRV-C clades show different recombination events and these are denoted individually as HRV-C.I to IV.

For the 632 HRV and HEV sequences from Latin America, separate phylogenetic trees were inferred for two regions: the 5’UTR and the VP4/VP2 protein-coding region (Figure 6). A lack of detectable spatial patterns in the data is shown for the VP4/VP2 coding region (upper left tree), with all viruses detected in all countries (data not shown).

The RDP, BOOTSCAN, and GENECONV algorithms available in the RDP were used to identify recombinant viruses within the coding and 5’UTR regions, separately. No recombination events were detected within the coding regions, but recombination was observed in the 5’UTR region for eight rhinoviruses (by at least two of the three methods, p < 0.05).

Recombination between the coding and 5’UTR regions was identified by inferring separate phylogenies for the coding and 5’UTR sequences and identifying major incongruencies in tree topology.

The lower right tree depicting the 5’UTR region shows that recombination events occur and their outcomes are circulating in this region. HRV-C frequently acquired the 5’UTR region of HRV-A by recombination processes, including multiple different clades, but this was not observed with HRV-B or any of the HEVs. Members of the HEV-A and HEV-B clades also seemed to undergo recombinant events at the 5’UTR region.

The HRV-C recombinant strains accounted for the majority of HRV-C viruses detected (Table 2) as only clade I contained the HRV-C with no recombination events. In Figure 5, 5’UTR analysis, we have labeled four HRV-C clades as they show different recombination events. We show one previously published isolate that is most representative of each clade (Table 2) [6,17,27,28] which allowed placement of known isolates like the NAT045 isolate in clade HRV-C.II and the Antwerp HRV 98/99 isolate in clade HRV-C.IV to better understand the variability of the HRV-C strains and to compare to other typing proposals [29].

**Table 2:** HRV-C clades 5’UTR phylogenetic analysis

**Clade**	**% of Total HRV C samples**	**Genbank accession number**	**Country of collection**	**Year of collection**	**Strain identifyer**	**Reference**
I	29	GQ223228	China	2007	N10	Huang et al. (2009) [17]
II	24	EF077280	USA	2003	NAT045	Kistler et al. (2007) [27]
III	18	AB683895	Phillipines	2011		Fuji et al. (2011) [6]
IV	29	JN990702	USA	2009	26	Lau et al. (2007) [28]

The 5’UTR phylogenetic tree enable us to define four clades of HRV-C serotype (in Figure 6). The percentage of HRV-C samples included in each clade is shown. For each clade, we included one previously published isolate that is most representative of each clade, which allowed placement of known isolates like the NAT045 isolate in clade HRV-C.II and the Antwerp HRV 98/99 isolate in clade HRV-C.IV to better understand the variability of the HRV-C strains and to compare to other typing proposals [29]. Each GenBank isolate accession number, country of collection and year of collection, strain identifier, and the reference manuscript are shown.

## Discussion

The frequency of HRV identified in our subjects with ILI is similar or greater to what has been found for influenza virus [30,31], human metapneumovirus [32,33], respiratory syncytial virus [30,31], and adenovirus [31,34] in the region, emphasizing the potential clinical and public health importance of this virus. Although differences in subject recruitment strategies prevent exact comparison between our studies and others, our HRV detection proportions are similar to pediatric respiratory disease studies from North America and Asia which had proportions that varied between 7.7% and 17.4% [7,17,35]. In South America, three separate studies from Brazil observed HRV detection proportions with a broader range, 15.9% to 46.7%, in children with ARI [22,23,25].

To further characterize the genetic diversity of these viruses, we sequenced all HRVs and HEVs identified. HRV-A and HRV-C species accounted for the majority of the HRV viruses (58% and 38%, respectively), and showed, in both cases, a great variety of serotypes, including more than 60 for HRV-A. The relative low percentage of HRV-B detected (4% of total HRVs) could relate to the fact that this virus has been shown to present with no fever [7] and our inclusion criteria required a fever ≥38°C, and thus, we could have underestimated the presence of this virus in the population. However, others have noted similar compositions of the three HRV species, with HRV-B accounting for only 4-11% of all HRVs recovered from subjects with respiratory complaints [7,17,22,35]. Among HEVs, coxsackieviruses, both A and B, were the most predominant. We also identified low numbers of EV-D68, which has been associated with respiratory disease by others [10], although never in the countries of our study. In addition, we identified two cases of EV-A71, an agent traditionally associated with hand-foot-and-mouth disease, as well as severe neurological and cardiac complications [36,37]. EV-A71 has also been infrequently linked with respiratory disease in Canada [38], Taiwan [39], and Australia [40], although never in Latin America. While the prior reports were associated with focal EV-A71 outbreaks, no outbreak in Peru was reported during our study period. Nevertheless, the fact our surveillance spanned multiple Latin American countries over a one-year period and the only two EV-A71 cases occurred within two weeks of each other in towns separated by a mere 25 kilometers suggests a possible unidentified localized outbreak. Other similarities between our EV-A71 respiratory cases and those of prior reports include age < 5 years and mild symptoms.

Our study shows that children younger than 1 year had a higher proportion of HRV infection than older children and young adults, which is consistent with previous studies [7,19,41]. Children 5 years or younger had a slightly higher risk of infection by HRV-C compared with those older than 5. However, no differences in the prevalence among age groups were detected for HEVs throughout our network.

A second virus was detected in approximately 11% of HRV-positive samples, much lower than a rate (37%) reported by others [23]. We used two methods of detection, PCR for influenza viruses and cell culture for all other viruses (including influenza virus), perhaps allowing for higher identification proportions of influenza and most likely underestimating the number of secondary infections. However, we did not observe more severe symptoms in patients with another respiratory virus identified, a finding that is consistent with what has been reported previously [23].

Several studies have suggested differences in illness severity between HRV species, including a specific association of HRV-C with wheezing and asthma exacerbation [6,7,35]. In agreement with these studies, our results showed a higher frequency of dyspnea in subjects with HRV-C compared with other viruses; in addition, we noted an association of HRV-C detection with pre-existing respiratory diseases, in particular with asthma.

Our phylogenetic analyses showed the presence of almost all known serotypes of HRV-A and HRV-B, as well as the presence of HRV-C. HRV-C variants are not yet formally classified into types, although there have been some proposed typing methods [29]. We show that recombinant events occur at the 5’UTR [8] consistent with others who reported picornaviruses having recombination breakpoints restricted to non-structural regions of their genome [42]. We showed that the circulating HRV-C recombinants accounted for the majority of all HRV-C species, illustrating how much this virus varies over a very short period of time.

Limitations of our study include a lack of uniformity in samples collected among countries and the fact that we collected samples over a period of only one year, preventing definitive characterization of the seasonality of HRV and HEV in Latin America. Although an analysis spanning three to five years would have been ideal, we analyzed the temporal distribution of the three HRV species and HEVs by aggregating collection sites by their climatic and geographic similarities. As has been previously described in other continents [13,43], HRVs and HEVs showed a similar “year-long” temporal distribution throughout Central and South America during the one-year period of collection. We did not detect any seasonality for specific HRV-A or HRV-B species, although HRV-C species seemed to possess opposite seasonal trends on either side of the equator. Others have noted seasonal differences among the individual HRV species, including high rates of HRV-A in April in the U.S. [35] and low rates in summer in China [41], high rates of HRV-B during winter in Australia [40] and China [41], and high rates of HRV-C in October in the U.S. [35] and winter in China [17]; like ours, these studies collected samples for only a one or two-year time span. Another important limitation of our study was that the identification of HRV and HEV did not unequivocally imply causality, a factor that would have been obviated with the inclusion of age-matched asymptomatic controls.

Although we are not the first to demonstrate a high prevalence of HRV in children with ILI, our results provide new perspectives into this virus’s global reach and additional insight into its clinical and phylogenetic characteristics in this underreported area of the world. While proven preventive and treatment strategies exist for other common respiratory viruses, including respiratory syncytial virus (pharmacotherapy, passive immunization), adenovirus (active immunization), and influenza virus (pharmacotherapy, active immunization), no HRV vaccine or antiviral currently exists [44,45]. Our high prevalence of HRV and HEV identification in young Latin Americans indicates that pharmacotherapy against influenza virus or the common bacterial etiologies of respiratory disease should not be presumptive, but guided by diagnostic confirmation when available in this population. Despite the current limitations of rapid diagnosis and clinical management of HRV or HEV respiratory disease, we hope that surveillance such as ours will provide further impetus for the development of rapid diagnostic methods, vaccines, and antivirals, as well as further elucidation of what populations are most at risk and what types of HRV and HEV species are most worth targeting.

## Methods

### Ethics

This protocol was approved as less than minimal risk research by the Naval Medical Research Center (NMRC), Silver Spring, Maryland. Institutional Review Board (IRB; Protocol NMRCD.2002.0019) in compliance with all applicable federal regulations governing the protection of human subjects. Authorization was given to perform the study using an information sheet approved and stamped by the IRB. As this was part of clinical care and routine surveillance benefiting the ministries of health, verbal consent was obtained from all participants. This method of consent was accepted by the NMRC IRB as well as by each of the institutions involved.

### Specimen and data collection

From September 2010 to September 2011, in collaboration with eight Central and South American countries’ ILI passive surveillance networks (Nicaragua, El Salvador, Venezuela, Colombia, Ecuador, Peru, Paraguay and Argentina; Figure 1), nasopharyngeal swabs were collected from 3,375 subjects with ILI presenting to outpatient medical clinics in 21 cities, as described previously [46-49]. All participants were 25 years old or younger, had a fever (≥38°C), and either a cough or sore throat [50]. Once collected, swabs were placed in viral transport media and stored at −80°C until they were transported on dry ice to the Naval Medical Research Unit No. 6 (NAMRU-6) facilities in Lima, Peru. Every site used an identical case report form. This form collected basic epidemiologic information and symptoms prior to presentation.

### DNA extraction and PCR

We extracted and amplified viral RNA from 140 μl of the viral transport media using a viral RNA kit (QIAamp, Qiagen®). This was performed in 22 μl of reaction mixture consisting of 2.2 μl of nuclease-free water, 3 μl of MgSO_4,_ (5.0 ×), 15 μl of 2× reaction mix (Super Script III One- Step RT-PCR System) with platinum (Taq High Fidelity kit), 0.6 μl 20 μM concentrations of the sense and antisense primers, 0.6 μl Enzyme Mix, and 8 μl of template. HRV and HEV detection was performed by semi-nested reverse transcription–PCR (RT-PCR) targeting the 5’UTR and a partial sequence of the VP4/VP2 genes including the nucleotides 165–1079 of the genomic RNA. We used P1-1 HRV (CAAGCACTTCTGTYWCCCC) and 9565_R HRV (GCATCNGGYARYTTCCACCACCAICC) [12]. The amplification was carried out in a thermocycler 7700 (Applied Bioystems, Foster City, CA). Cycling conditions included a reverse transcription step at 50°C for 30 min and 95°C for 15 min followed by 45 PCR cycles: 94°C for 30 seconds; 55°C for 30 seconds; 72°C for 90 sec; and final incubation for 72°C for 10 min. The amplified products of 915 bp were analyzed by electrophoresis on an agarose 2% gel. PCR products were purified with Centri-Seps Columns (Princeton Separations). Purified products were directly used for sequencing of viral nucleic acids from clinical specimens.

### Sequencing and phylogenetic analyses

The 5’UTR and VP4/VP2 coding region of all HRV and HEV positive samples obtained (n = 632) were sequenced and included in the following phylogenetic analyses. These regions were selected for sequencing because VP4/VP2 is the most commonly studied region of HRV and the 5’UTR regions have been shown to recombine frequently. For direct sequencing of viral nucleic acids from clinical specimens, gene fragments were amplified and sequenced with the use of a Big Dye terminator cycle sequencing kit (version 3.1, Applied Biosystems) and internal primers Generic F HRV (AGCCTGCGTGGCKGCC) and NCR2 HRV (ACTACTTTGGGTGTCCGTGTTTC) on a Genetic Analyser system (version 3130xL, Applied Biosystems).

Separate alignments of the coding (VP4/VP2; 464 nt) and non-coding (555 nt) regions’ sequences were constructed using MUSCLE v.3.8.31 [51]. Maximum likelihood phylogenetic trees were inferred separately for the non-coding and coding regions using PhyML v.3.0 using a general-reversible substitution model with gamma-distributed among-site rate variability [52]. Phylogenetic trees were visualized in FigTree v1.3.1 and colored by HRV species: HRV-A, HRV-B, HRV-C, and four clades of HEV (HEV-A, HEV-B, HEV-C, and HEV-D). Complete sequences (≈1,000 nt) were assembled, aligned, and edited using Sequencer (version 4.8 – Gene Codes Corporation, Ann Arbor, MI, USA) and BioEdit (version 7.0.0 -Isis Pharmaceuticals, Inc., Dublin, Ireland) software. Phylogenetic trees were generated with CLUSTAL X version 2.0.1 and MEGA version 3.1 software [53]. Fifty-eight HRV and 34 HEV sequences, representing all different clades found during the analysis were submitted to GenBank and their accession numbers are: JX129393 - JX129484. The RDP, BOOTSCAN, and GENECONV algorithms available in the Recombination Detection Program (RDP3, available at http://web.cbio.uct.ac.za/~darren/rdp.html) were used to identify recombinant viruses within the coding and 5’UTR regions, separately.

### Detection of other respiratory viruses

All HRV- and HEV-positive samples underwent viral isolation by inoculation into four cell lines: MDCK, Vero76, VeroE6 and LLCMK2 cells (ATCC, Manassas, VA 20108), as previously reported [54]. After ten days of culture, the cells were spotted onto microscope slides. Cell suspensions were dried and fixed in chilled acetone for 15 minutes. Immunofluorescence assay was performed using the Respiratory Virus Screening and Identification Kit (D3 DFA Respiratory Virus Diagnostic Hybrids; Athens, OH) for the identification of adenoviruses, influenza A and B viruses, parainfluenza viruses (types 1, 2, 3 and 4), and respiratory syncytial virus.

In addition, real time-PCR for influenza viruses was performed on all HRV- and HEV-positive samples as previously described [49]. This was performed as part of the routine sample processing of the respiratory surveillance network at NAMRU-6.

### Statistical analyses

Data was entered into a database using Microsoft Access and analyzed using Stata/SE 10.0 for Windows (StataCorp LP, College Station, TX). Two proportion z-tests were used to compare proportions; p-values ≤0.05 were considered statistically significant; 95% confidence intervals (C.I.) were calculated for each odd ratio (O.R.), and associations were assessed using Pearson’s chi-square (*×*^*2*^*)* or Fisher’s tests.

## Competing interests

None of the authors has a financial or personal conflict of interest related to this study. The corresponding author had full access to all data in the study and final responsibility for the decision to submit this publication.

## Authors’ contributions

JG and TJK contributed in the design and conception of the study. VE, MS and MVV performed the analysis of the data. JG, ESH and MN performed the analysis and interpretation of data. JG drafted and submitted the manuscript. JG, MB, FS, GC, AEA, NA, ILR, WC, MJ, WA, FR and MG contributed with the acquisition of data. All authors contributed with the critical revision and final approval of manuscript.

## Copyright statement

Authors Tadeusz J. Kochel and Eric S. Halsey are military service members and Josefina Garcia, Manuel Villaran, and Victoria Espejo are employees of the U.S. Government. This work was prepared as part of their official duties. Title 17 U.S.C. § 105 provides that ‘Copyright protection under this title is not available for any work of the United States Government’. Title 17 U.S.C. § 101 defines a U.S. Government work as a work prepared by a military service members or employees of the U.S. Government as part of those person’s official duties.
